# MET inhibitors for targeted therapy of EGFR TKI-resistant lung cancer

**DOI:** 10.1186/s13045-019-0759-9

**Published:** 2019-06-21

**Authors:** Qiming Wang, Sen Yang, Kai Wang, Shi-Yong Sun

**Affiliations:** 1 Department of Internal Medicine, The Affiliated Cancer Hospital of Zhengzhou University, Henan Cancer Hospital, Zhengzhou, China; 2 Department of Respiratory Medicine, Second Affiliated Hospital, Zhejiang University School of Medicine, Hangzhou, China; 3 Department of Hematology and Medical Oncology, School of Medicine and Winship Cancer Institute, Emory University, 1365-C Clifton Road, C3088, Atlanta, GA 30322 USA

## Abstract

Treatment of non-small cell lung cancer (NSCLC) harboring epidermal growth factor receptor (EGFR) activating mutation with EGFR-TKIs has achieved great success, yet faces the development of acquired resistance as the major obstacle to long-term disease remission in the clinic. *MET* (or *c-MET*) gene amplification has long been known as an important resistance mechanism to first- or second-generation EGFR-TKIs in addition to the appearance of T790 M mutation. Recent preclinical and clinical studies have suggested that *MET* amplification and/or protein hyperactivation is likely to be a key mechanism underlying acquired resistance to third-generation EGFR-TKIs such as osimertinib as well, particularly when used as a first-line therapy. EGFR-mutant NSCLCs that have relapsed from first-generation EGFR-TKI treatment and have *MET* amplification and/or protein hyperactivation should be insensitive to osimertinib monotherapy. Therefore, combinatorial therapy with osimertinib and a MET or even a MEK inhibitor should be considered for these patients with resistant NSCLC carrying *MET* amplification and/or protein hyperactivation.

## Introduction

Lung cancer is the leading cause of cancer death among both men and women and accounts for one third of all cancer deaths worldwide. Non-small cell lung cancer (NSCLC) constitutes over 80% of lung cancer cases and has a low 5-year survival rate of about 18% [1], despite great efforts made worldwide over the past decades to combat lung cancer. The development of epidermal growth factor receptor (EGFR) tyrosin1e kinase inhibitors (EGFR-TKIs) based on the discovery of EGFR-activating mutations is an important milestone in the targeted therapy of NSCLC.

The majority of EGFR-activating mutations (~ 90%) primarily present as an exon 19 deletion (Del19; ~ 60%) or exon 21 point mutation L858R (~ 30%). The prevalence of these mutations is ~ 15% and ~ 40% in Western and Asian populations with NSCLC, respectively [2]. These EGFR mutations increase the affinity of EGFR-TKIs for the mutant receptor, thus conferring sensitivity to EGFR-TKI treatment. First-generation EGFR-TKIs, such as gefitinib and erlotinib, are competitive reversible inhibitors of ATP, thereby preventing autophosphorylation of the TK domain and blocking the activation of signaling downstream of EGFR [2]. First-generation EGFR-TKIs provide significant clinical benefit in patients with these mutations, representing the first successful targeted therapy against lung cancer. However, patients eventually develop disease progression because of acquired resistance, which limits the long-term efficacy of these agents [2–4].

Acquired resistance to first-generation EGFR-TKIs is often caused by the acquisition of the T790 M mutation, which accounts for approximately 60% of resistant cases. In addition, *MET* (*c-MET*) gene amplification is another important mechanism and is detectable in approximately 5–22% of NSCLC patients with acquired resistance to first-generation EGFR-TKIs [2–4]. Mechanistically, *MET* amplification causes EGFR-TKI resistance by activating EGFR-independent phosphorylation of ErbB3 and downstream activation of the PI3K/AKT pathway, providing a bypass pathway in the presence of an EGFR inhibitor. This redundant activation of ErbB3 permits cells to transmit the same downstream signaling in the presence of EGFR-TKIs. Thus, concomitant inhibition of both EGFR and MET would be required to overcome resistance to EGFR inhibitors by *MET* amplification [5]. Although *MET* amplification can occur with the EGFR T790 M mutation, about 60% of *MET* amplification is found without T790 M mutation. There is an inverse correlation between the presence of T790 M and *MET* gene copy number, suggesting a complementary or independent role of the two mechanisms in the acquisition of resistance [6].

**Fig. 1:**
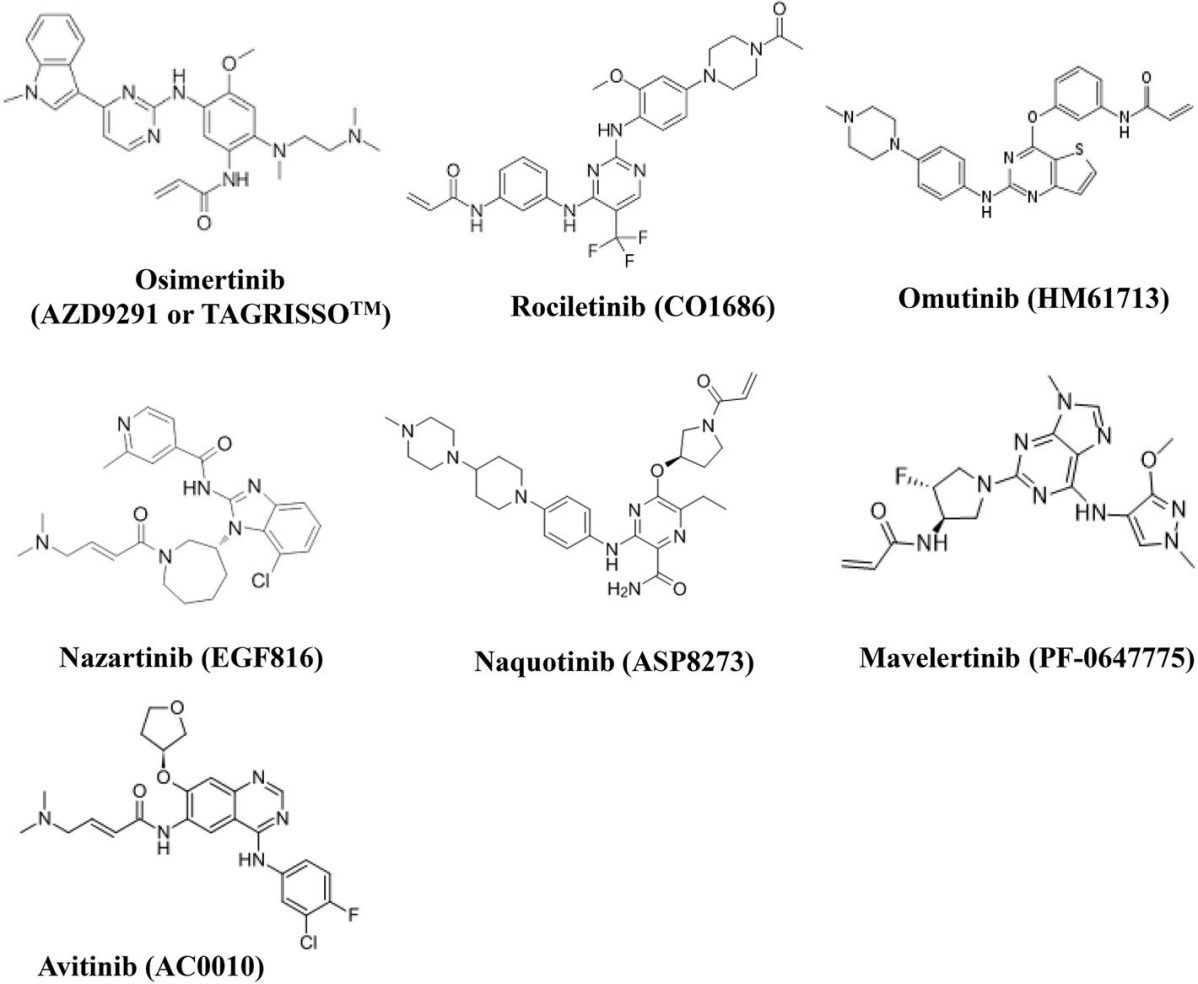
Chemical structures of third-generation EGFR-TKIs

Osimertinib (AZD9291 or TAGRISSO^TM^), rociletinib (CO1686), olmutinib (HM61713), nazartinib (EGF816), naquotinib (ASP8273), mavelertinib (PF-0647775), and avitinib (AC0010) are examples of third-generation EGFR-TKIs, which selectively and irreversibly inhibit the common “sensitive” EGFR mutations, Del19 and L858R, and the resistant T790 M mutation while sparing wild-type (WT) EGFR (see their chemical structures in Fig. 1). Osimertinib is now an FDA-approved drug for treating patients with NSCLC that has become resistant to the first-generation EGFR-TKIs through the T790 M mutation and for EGFR mutation-positive advanced NSCLC as a first-line treatment. Although osimertinib has achieved great success in the clinic, all patients have eventually relapsed and developed resistance to the treatment, resulting in treatment failure. Unfortunately, the resistance mechanisms are largely unknown except for some related to C797S mutation and *MET* amplification.

To conquer resistance to EGFR TKIs, many clinical trials that test novel EGFR, MET, and VEGFR inhibitors have been designed and launched in China and all over the world [7–9]. Toward C797S mutation, the fourth-generation EGFR-TKIs such as EAI045 has been developed and is under preclinical development [10]. This review will primarily focus on the role of *MET* amplification in mediating acquired resistance to osimertinib as well as other third-generation EGFR-TKIs.

## MET structure and function

**Fig. 2:**
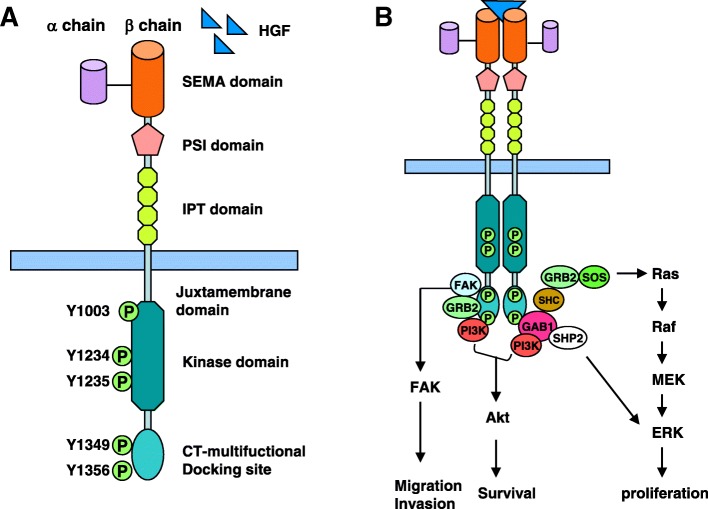
MET protein structure (**a**) and HGF/MET signaling pathway (**b**). GRB, growth factor receptor-bound protein; SHC, Src homology 2 domain-containing; PI3K, phosphatidylinositol 3-kinase; SOS, son of sevenless; SHP2, Src homology region 2-containing protein tyrosine phosphatase 2; FAK, focal adhesion kinase

*MET* proto-oncogene exists in the long arm of human chromosome 7 and encodes MET (c-MET) protein that is a membrane tyrosine kinase receptor. The initially encoded preproprotein is proteolytically processed to generate α and β subunits that are linked via disulfide bonds to form the mature receptor. The binding of MET to its ligand, hepatocyte growth factor (HGF) secreted by stromal cells, induces dimerization and activation of the receptor. Therefore, the activated MET is a heterodimer linked by an extracellular α chain and a transmembrane β chain that contains a SEMA (sema homology region) domain, a PSI (plexin-semaphorin-integrin) domain, four IPT (immunoglobulin-like regions in plexins and transcription factors) domains, a transmembrane domain, a juxtamembrane domain, a tyrosine kinase domain, and a C-terminal tail region. The SEMA domain is the site where HGF binds directly to MET, and PSI can stabilize this interaction. When HGF binds MET, autophosphorylation of Y1234 and Y1235 in the intracellular tyrosine kinase domain occurs, resulting in Y1349 and Y1356 autophosphorylation in the C-terminal multifunctional docking site. This induces the recruitment of several intracellular effector adaptor proteins such as growth factor receptor-bound protein 2 (GRB2), GAB1, SRC, and PI3K and consequently the activation of downstream signaling pathways (Fig. 2) [11, 12]. The HGF/MET signaling pathway is highly regulated and plays an important role in cell proliferation, survival, embryogenesis, and cellular migration and invasion [11–13]. The main types of variation of HGF/MET signaling pathway in NSCLC patients are point mutations, amplification, exon 14 skipping mutations, and fusion [14].

## Preclinical studies demonstrating the role of MET amplification and protein hyperactivation in conferring resistance to third-generation EGFR-TKIs

The initial preclinical link between MET and resistance to third-generation EGFR-TKIs came from our observation that an EGFR mutant (EGFRm) NSCLC cell line with acquired resistance to erlotinib (HCC827/ER), which has amplified *MET* gene and hyperactivated MET protein, was cross-resistant to both osimertinib and rociletinib. Moreover, HCC827 cells with acquired resistance to osimertinib (HCC827/AR) also possessed amplified *MET* gene and hyperactivated MET protein, evidenced by increased p-MET levels in comparison with the parental cell line, and were resistant to not only rociletinib, but also erlotinib [15]. In agreement, inhibition of MET with either a small molecule MET inhibitor or a genetic knockdown of *MET* expression restored the ability of osimertinib to effectively inhibit the growth of both HCC827/ER and HCC827/AR cells in vitro and in vivo and to inactivate ErbB3 or suppress ErbB3 phosphorylation [15]. Our findings together suggest that *MET* gene amplification and protein hyperactivation are likely a common resistance mechanism to both first- and third-generation EGFR-TKIs. Moreover, our results also suggest that monotherapy with osimertinib or other third-generation EGFR-TKIs will likely be ineffective for the treatment of EGFRm NSCLCs with acquired resistance to first-generation EGFR-TKIs due to *MET* amplification and/or protein hyperactivation.

Consistently, several recent studies have generated similar observations. HCC827 cells resistant to erlotinib, which were established in a different laboratory, exhibited *MET* amplification with increased protein expression and were resistant to osimertinib [16]. Similarly, H1975-P1 cells resistant to AC0010 derived from resistant H1975 xenografts in nude mice after treatment for over 3 months or selection with AC0010 possessed overexpressed *MET* gene with increased levels of both MET protein and p-MET and were cross-resistant to afatinib, osimertinib, and rociletinib [17]. In a naquotinib-resistant clone (PC-9/NaqR2) derived from the EGFRm PC-9 cell line, *MET* amplification was also detected accompanied with elevated levels of both MET and p-MET. This resistant line was cross-resistant to gefitinib but sensitive to the combination of naquotinib with a MET inhibitor (crizotinib or SGX532) [18].

## Detection of MET dysregulation in clinical cancer tissue specimens or circulating tumor DNA

MET dysregulation in human cancer tissues can be detected at the gene level (e.g., amplification) and at the protein level as discussed below. Beyond, MET alterations in ctDNA should be another way for detecting the dysregulation (Table 1).

**Table 1:** Assays for the detection of MET dysregulation

Methods	Principle	Criterion	Specialty
FISH	The MET gene copy numbers were obtained by detecting the sites of MET and CEP7 (as the control).	1. The ratio of MET vs. CEP7: low amplification (≥ 1.8, < 2.2), medium amplification (> 2.2, <5), and high amplification (≥ 5). 2. The proportion of positive cells in total cells.	Advantages: high accuracy; good repeatability; good correlation with the curative effect, and less specimens can be detected.
			Disadvantages: fluorescence microscopy equipment and experienced operator are required; MET expressed on the cell surface but not amplified could not be detected.
ddPCR	Detecting the difference in fluorescence signal strength between the amplificated MET site and internal reference site.	MET gene amplification was defined by ddPCR as MET copy number > 5.5	Advantages: high accuracy and high detection speed.
			Disadvantages: high requirement for DNA fragment quality.
IHC	Anti-c-MET (SP44) rabbit monoclonal antibody was used as the primary antibody and positive results were determined by evaluating the staining status of the cells. 2+ or 3+ is defined as high MET expression, and 0 or 1+ is defined as low MET expression (Metmab criteria).	3+ (≥ 50% tumor cells strongly positive), 2+ (≥ 50% tumor cells positive/weakly positive or < 50% tumor cells strongly positive), 1+ (weakly positive tumor cells ≥ 50% or positive cell number < 50%), and 0 (the number of tumor cells without staining or with any intensity staining < 50%).	Advantages: mature technology, rapid and simultaneous results in many cases, simultaneous observation of cell morphology, and low cost.
			Disadvantages: the result interpretation is subjective; easy to be disturbed in the testing process.
NGS	CNV can be estimated by calculating the coverage (sequencing depth) of the region where the MET gene is located. The coverage area is divided into a continuous bin, and the final copy number given is the average of all bin of a gene.	The covering depth of more than 60% of the bin of a gene in cancer samples is significantly higher than the baseline level (*z* test), and the covering level of the entire gene region is statistically significant different from the baseline level (*t* test); the cutoff taken by different algorithms is different.	Advantages: multi-gene parallel detection can be achieved by tissue or blood detection, and all mutation, deletion, amplification, fusion, and other mutation types can be detected at one time, with high detection sensitivity.
			Disadvantages: high testing cost, need NGS sequencing equipment, and high technical requirements.

*Abbreviation*: *MET* mesenchymal-epithelial transition factor, *FISH* fluorescence in situ hybridization, *CEP7* centromeric region of chromosome 7, *ddPCR* droplet digital PCR, *IHC* immunohistochemistry, *NGS* next-generation sequencing, *CNV* copy number variation

### MET amplification

The increase in copy number of the MET gene can occur in both polyploidy and amplification. Polyploidy is the duplication of chromosomes, and multiple copies of chromosome 7 are present in tumor cells. Polyploidy is not a driving gene in biology. Amplification is the duplication of local or regional genes, and the fault-fusion-bridge mechanism is the main cause of gene amplification. Compared with polyploidy, MET amplification may serve as a driving gene and is one of the main mechanisms of EGFR-TKIs resistance. The MET gene copy number is a continuous variable, and the definition of a positive threshold affects the incidence, the rate of overlap with other genotypes, and the ability to predict the efficacy of MET inhibitors [19].

MET amplification can be detected using a FISH method that detects the MET/CEP7 value to distinguish polyploidy from amplification. In polyploidy, MET copy genes have corresponding centromeres, and MET/CEP7 values do not change despite an increase in the number of MET copies. NGS can also be used for amplification detection and requires comparison with normal diploid. There is no consensus on how many copies of the gene are MET positive. Current practice divides MET/CEP7 into low-level amplification (1.8, < 2.2), medium-level amplification (> 2.2, < 5), and high-level amplification (≥ 5) [19].

### MET overexpression

MET overexpression can be caused by gene amplification, gene mutation, and transcriptional enhancement or by post-transcriptional mechanisms. IHC can be used to detect MET overexpression in tissue specimens. Due to differences in antibodies and thresholds, the proportion of MET overexpression in NSCLCs varied greatly in different studies, ranging from 15 to 70%. The proportion of MET overexpression was much higher than that of MET mutation and amplification.

### MET alterations in ctDNA

Beyond tissue specimen, a recent study using digital sequencing of ctDNAs from 438 patients analyzed clinical associations of MET alterations in the plasma of patients with diverse malignancies including NSCLC and showed that MET ctDNA alterations were associated with a poorer prognosis, higher numbers of genomic abnormalities, and bone metastases. This study has demonstrated that detection of MET alterations by liquid biopsy is feasible. MET alterations were observed in 7.1% patients, which is higher than in the frequency in tissues (1.14%; *P* = 0.0002) [20].

## Clinical detection of MET amplification in EGFRm NSCLCs relapsed from treatment with third-generation EGFR-TKIs

In line with our preclinical findings, clinical detection of *MET* amplification in EGFRm NSCLCs after relapse from osimertinib or other third-generation EGFR-TKIs was also reported. An early case report documented that a patient who developed resistance to osimertinib after a confirmed partial response for 9 months had a high level of *MET* amplification post-osimertinib treatment [21]. This observation has been subsequently confirmed by several clinical studies with different cohorts of patients although the frequencies of *MET* amplification have varied.

While only one case of *MET* amplification (4%) was detected among 25 NSCLC patients positive for EGFR T790 M that developed resistance to osimertinib [22], other studies have detected much higher frequencies of *MET* amplification. Le et al. [16] reported that 5 cases of *MET* amplification (14%) were detected among 42 cases of progression following treatment with osimertinib. Piotrowska et al. [23] analyzed tissue biopsies from 32 osimertinib-resistant EGFRm NSCLC patients and detected 7 (22%) carrying *MET* amplification, but only 6 patients (19%) with acquired *EGFR* C797S. Another study by Oxnard et al. [24] documented that among 41 patients who developed resistance to osimertinib and underwent biopsy after relapse, 4 cases (10%) of *MET* amplification were detected. Analysis of plasma samples from 73 patients with resistance to osimertinib second-line treatment in the large phase III clinical study AURA3 showed that *MET* amplification was the most common (19%) resistance mechanism, followed by EGFR C797 secondary mutation (15%), with 10 cases of C797S and 1 case of C797G [25].

In a cohort of Chinese NSCLC patients with T790 M enrolled in the AURA trial, 5 (50%) of 10 patients assessed for *MET* amplification were positive; however, C797S was detected only in two (17%) of 12 assessed patients [26]. In a different study with a cohort of 13 Chinese NSCLC patients who developed disease progression after osimertinib, 4 cases of *MET* amplification (31%) were detected [27]. Interestingly, no *MET* amplification was detected through core needle biopsy and next-generation sequencing (NGS) in another cohort of 9 Chinese patients after progression with osimertinib treatment although C797S or C797G was detected in 5 patients [28]. Another study with a cohort of 93 Chinese NSCLC patients relapsed from osimertinib treatment reported 5 cases of *MET* amplification (5.4%). Moreover, two other cases containing rare mutations of MET P97Q and I865F were also detected although the biological functions of these mutations are unknown [29].

Similar observations have been made in studies with other third-generation EGFR-TKIs. Although the EGFR C797S mutation was initially detected in ∼ 32% of patients after relapse from osimertinib treatment [30], analysis of circulating tumor DNA (ctDNA) in 43 EGFRm NSCLC patients resistant to rociletinib treatment revealed < 3% EGFR C797S mutation, but increased *MET* copy number in 11 patients (26%) [31]. In a more recent study, *MET* amplification was observed only in 7.6% (5/66) of patients with acquired resistance to rociletinib [32]. Consistently, a low percentage of C797S mutation (4.5%; 3/66) was detected in this study. In a study of 16 EGFRm NSCLC patients with development of resistance to AC0010, *MET* amplification was detected in only one case (6.25%), but EGFR C797S mutation was not detected [33].

The majority of studies reported so far have focused on the development of resistance to osimertinib or other third-generation EGFR-TKIs as second-line treatment. Information regarding *MET* amplification in resistance to first-line osimertinib treatment for EGFR mutation-positive advanced NSCLC is limited, largely due to its recent approval for this indication. In the first reported trial, *MET* amplification was detected in one case (5.3%) among 19 patients with detectable circulating plasma tDNA [34]. In a study analyzing 91 plasma samples by NGS from patients receiving first-line treatment with osimertinib in the phase III FLAURA clinical trial recently presented at the 2018 ESMO annual meeting, the most common acquired resistance mechanism was *MET* amplification (15%) followed by EGFR C797S mutation (7%) [35].

## Therapeutic strategies for treating EGFRm NSCLCs resistant to first- or second-generation EGFR-TKIs due to MET amplification and for overcoming MET-mediated acquired resistance to third-generation EGFR-TKIs

Our preclinical studies suggest that monotherapy with osimertinib or other third generation EGFR-TKIs will likely be ineffective for the treatment of EGFRm NSCLCs with acquired resistance to first- or second-generation EGFR-TKIs due to *MET* gene amplification and protein hyperactivation [15]. In the clinic, patients with multiple pre-existing mechanisms (T790 M and MET) experienced inferior responses [31]. Moreover, patients with *MET* amplification after osimertinib resistance tended to have inferior median progression-free survival (PFS) and median overall survival (OS) than patients without the appearance of or increase in *MET* amplification [27]. Therefore, we need effective strategies for the treatment of patients with *MET*-amplified NSCLC that has relapsed from first- or second-generation EGFR-TKI treatment or patients who develop acquired resistance to osimertinib due to *MET* amplification and protein hyperactivation.

**Fig. 3:**
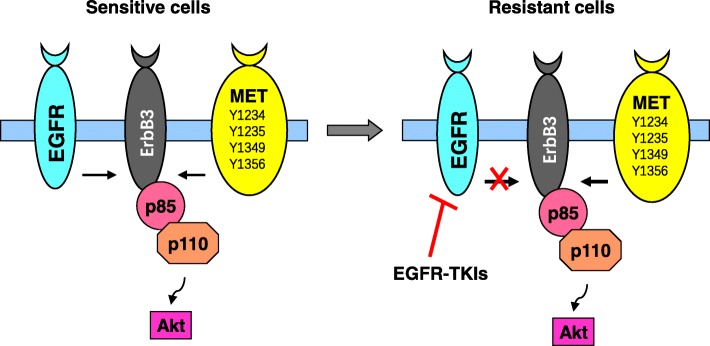
*MET* amplification causes EGFR-TKI resistance by activating EGFR-independent phosphorylation of ErbB3 and downstream activation of the PI3K/AKT pathway, providing a bypass resistance mechanism in the presence of an EGFR-TKI. MET can also activate PI3K/Akt signaling through ErbB3. In EGFRm NSCLCs with *MET* amplification, EGFR-TKIs can still inhibit EGFR phosphorylation but not ErbB3 phosphorylation, leading to persistent activation of PI3K/Akt signaling via ErbB3 in an EGFR-independent manner

The basic mechanism by which *MET* amplification causes EGFR-TKI resistance is associated with the activation of EGFR-independent phosphorylation of ErbB3 and downstream activation of the PI3K/AKT pathway, providing a bypass signaling pathway even in the presence of an EGFR-TKI (Fig. 3) [5]. Thus, co-targeting both EGFR and MET would be required to overcome resistance to EGFR-TKIs by MET amplification, as previously suggested [5]. Indeed, our preclinical studies have shown that inhibition of MET with either gene knockdown or small molecule MET inhibitor (e.g., crizotinib) combined with osimertinib very effectively inhibited the growth of HCC827/ER cells and HCC827/AR cells, which both have *MET* amplification, both in vitro and in vivo [15]. Similar results were also generated in different resistant models with *MET* amplification in different laboratories [17, 18, 31]. We found that ErbB3 phosphorylation in both HCC827/ER and HCC827/AR cell lines was minimally inhibited by osimertinib alone, but could be fully suppressed when combined with a MET inhibitor both in vitro and in vivo. This was also true for phosphorylation of other proteins including Akt, S6, and ERK1/2. Hence, full suppression of ErbB3 phosphorylation is tightly associated with the enhanced efficacy of osimertinib and its combination with MET inhibition against the growth of EGFR-TKI-resistant cell lines with *MET* amplification [15].

**Fig. 4:**
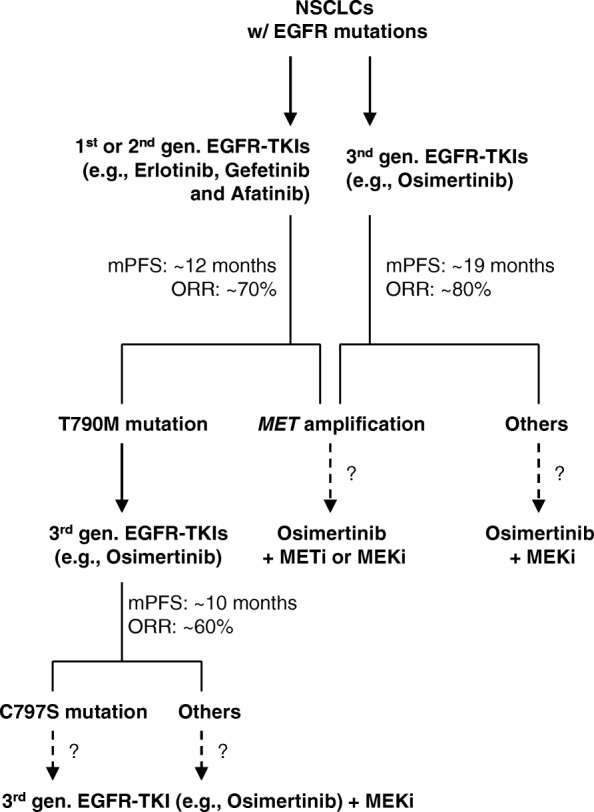
Current treatment options for EGFR-mutant NSCLCs and potential strategies for overcoming acquired resistance to osimertinib. The strategies as indicated with dashed lines need clinical validation. METi, MET inhibitor; MEKi, MEK inhibitor

A similar attempt has been made in the clinic. It was reported that combinatorial treatment of a first/third-generation EGFR-TKI and crizotinib was tested in two patients with newly acquired *MET* amplification after osimertinib resistance. Partial responses were achieved both clinically and radiographically [27]. A recent case report also shows that a patient with NSCLC harboring EGFR L858R mutation had emergent *MET* amplification after disease progression on erlotinib and had a sustained partial response to a combination of full-dose osimertinib and crizotinib with excellent tolerance [36]. Therefore, the current preclinical and clinical studies warrant further investigation of MET inhibition combined with osimertinib or other third-generation EGFR-TKIs for the treatment of EGFRm NSCLCs with *MET* amplification caused by treatment with first- or second-generation EGFR-TKIs or with third-generation EGFR-TKIs (Fig. 4).

Beyond MET inhibition as we discussed above, our recent preclinical studies suggest that MEK inhibition with a small molecule MEK inhibitor such as trametinib (GSK1120212) is also a very effective strategy in overcoming MET-mediated acquired resistance to osimertinib [37]. Different MEK inhibitors including trametinib, selumetinib (AZD6244), and PD0325901 were all very effective when combined with osimertinib in inhibiting the growth of HCC827/AR cells in vitro or tumors in vivo including induction of apoptosis [37]. The advantage of this therapeutic regimen over MET inhibition is its potent efficacy against not only osimertinib-resistant cells with *MET* amplification, but also other resistant cell lines with different underlying mechanisms including C797S mutation, which are not responsive to the combination of osimertinib and *MET* inhibition based on our results [37]. This is important in the clinic if this therapeutic strategy is active against acquired resistance to third-generation EGFR-TKIs regardless of their underlying mechanisms (Fig. 4).

## Clinical practice of MET inhibitors combined with an EGFR-TKI in the treatment of NSCLCs

**Fig. 5:**
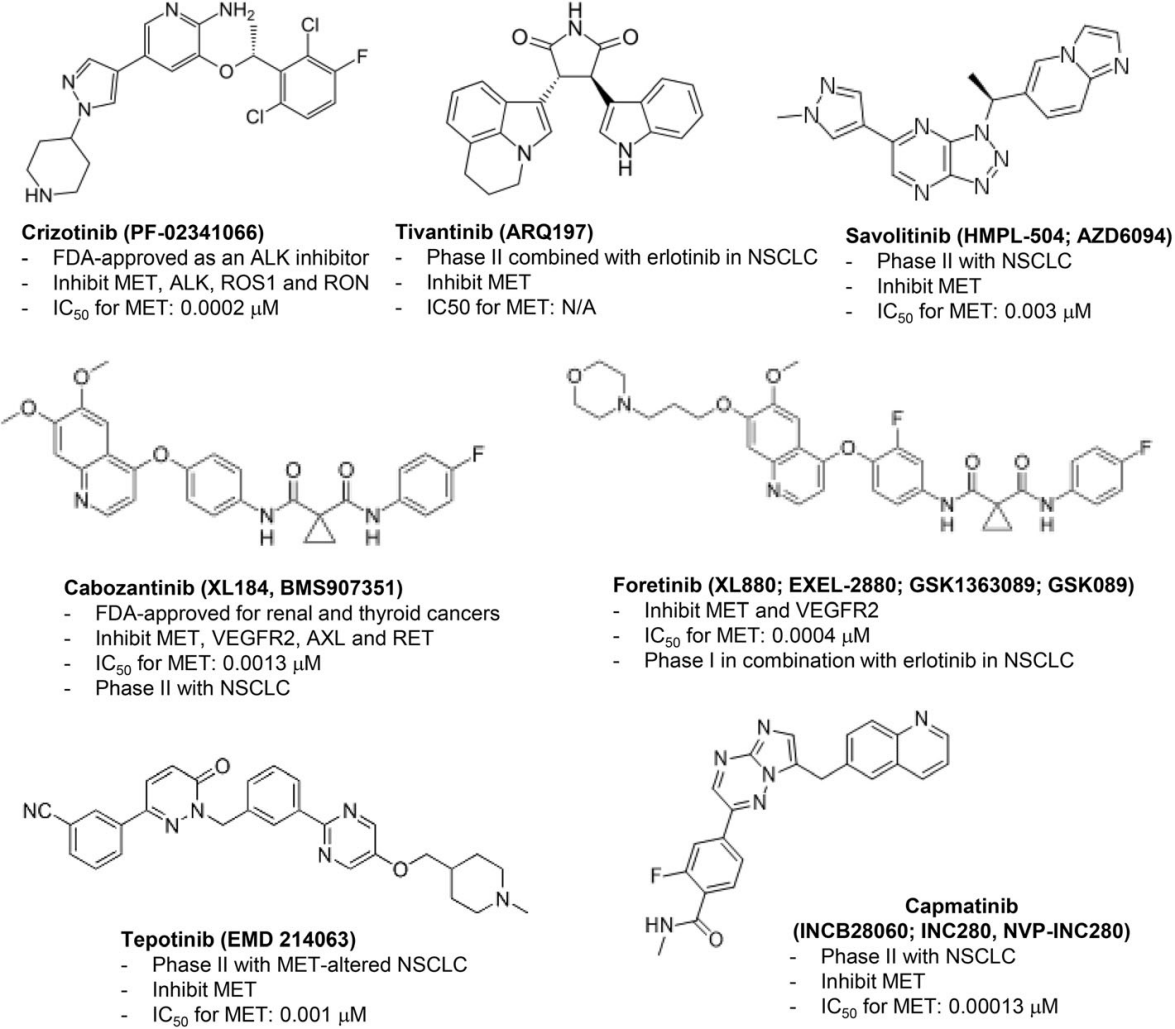
Chemical structures of small molecule MET inhibitors with their target specificities. IC_50_, half maximal inhibitory concentration; VEGFR2, vascular endothelial growth factor receptor 2; RET, rearranged during transfection; ALK, anaplastic lymphoma kinase; RON, Recepteur d'Origine Nantais

MET inhibitors can be divided into three categories: the small molecule MET receptor inhibitors (e.g., crizotinib, tivantinib, savolitinib, tepotinib, cabozantinib, and foretinib) (Fig. 5), the MET receptor monoclonal antibodies (e.g., onartuzumab), and antibodies against its ligand HGF (e.g., ficlatuzumab and rilotumumab) [38]. Some MET inhibitors have been tested in the clinic against NSCLCs combined with a first- or second-generation EGFR-TKI (Table 2). Mixed outcomes have been generated depending on whether patient populations were selected based on MET status. In general, these combinations did not show improved efficacies in the treatment of unselected NSCLC patient populations. However, some positive results were generated in patient populations selected for *MET* amplification or overexpression as highlighted below. Therefore, MET may still remain a rational target for therapy in patients with EGFR TKI-resistant and *MET*-amplified NSCLCs [39].

**Table 2:** Clinical trials testing the combination of a MEK inhibitor and an EGFR-TKI for the treatment of NSCLC patients

Study	NCT01244191	NCT01982955	NCT01610336	NCT01456325
Phase	III	II	II	III
Treatment arms	Tivantinib (360 mg twice a day) + erlotinib (150 mg once a day) vs. placebo (twice a day) + erlotinib (150 mg once a day)	Tepotinib (500 mg once a day) + gefitinib (250 mg once a day) vs. pemetrexed + cisplatin/carboplatin	Capmatinib (400 mg twice a day) + gefitinib (250 mg once a day)	Onartuzumab (15 mg/kg IV) + erlotinib (150 mg once a day) vs. placebo + erlotinib (150 mg once a day)
Patients (*n*)	1048	55	100	499
ORR (%)	10.3 vs. 6.5	66.7 vs. 42.9^a^	47^b^	8.4 vs. 9.6
PFS (months)	3.6 vs. 1.9 (HR = 0.74; *P* < 0.001)	21.2 vs. 4.2^a^	5.5^b^	2.7 vs. 2.6 (HR = 0.99; *P* = 0.92)
OS (months)	8.5 vs. 7.8 (HR = 0.98; *P* = 0.81)	NA	NA	6.8 vs.9.1 (HR = 1.27; *P* = 0.067)
Main grade 3 or higher toxicities (over 5%)	Fatigue or asthenia (9%), dyspnea (8.8%), and anemia (6.3%) in erlotinib plus tivantinib arm vs. fatigue or asthenia (7.9%) and dyspnea (7.4%) in erlotinib plus placebo arm	51.6% in tepotinib plus gefitinib arm and 52.2% in chemotherapy arm had grade ≥ 3 TRTEAEs	Nausea (5%), peripheral edema (5%), fatigue (6%), increased amylase (6%), and increased lipase (6%)	Overall skin and subcutaneous tissue disorders (17.3), rash (7.7%), and dyspnea (5.2%) in onartuzumab plus erlotinib arm vs. overall skin and subcutaneous tissue disorders (10.7%) and rash (5.3%) in erlotinib plus placebo arm

^a^In patients with MET gene amplification

^b^In patients with a MET gene copy number ≥ 6

### Tivantinib

In the MARQUEE phase III study, 1048 patients with advanced non-squamous NSCLC previously treated with one to two systemic regimens, including a platinum doublet, were randomly assigned in a 1:1 ratio to receive erlotinib plus tivantinib (E + T) or erlotinib plus placebo (E + P) until disease progression. OS was not improved with E + T versus E + P (median OS, 8.5 v 7.8 months) even though PFS increased (median PFS, 3.6 v 1.9 months). Exploratory subgroup analyses suggested OS improvement in patients with high MET expression [40]. It needs to be pointed out that NSCLC patients in this trial were not exclusively those with activating EGFR mutations and relapse from erlotinib treatment; the rationale for using erlotinib seemed not well justified.

### Tepotinib

At the 2018 ESMO conference, Dr. Wu’s group presented the first trial comparing the efficacy of tepotinib and gefitinib combination (T + G) with chemotherapy for EGFR+/MET + NSCLCs. In patients with *MET* amplification, the median PFS in the T + G group was more than five times longer than that in the chemotherapy group. In patients with *MET* amplification, T + G was 66.7% effective while chemotherapy was 42.9% effective in terms of response rate. Among patients with MET protein overexpression, the response rate of T + G was 68.4% versus 33.3% in the chemotherapy group. Subgroup analysis showed that patients with *MET* gene amplification receiving T + G treatment had a median PFS of 21.2 months, much longer than the 4.2 months among those receiving chemotherapy. T + G treatment was generally well tolerated [41].

### Capmatinib

In a recently reported phase Ib/II study investigating the safety and efficacy of capmatinib plus gefitinib in patients with EGFR-mutated, MET-dysregulated (amplified/overexpressing) NSCLC who experienced disease progression while receiving EGFR-TKI treatment, 61 patients were treated in phase Ib, and 100 were treated in phase II. Preliminary clinical activity was observed, with an overall response rate (ORR) across phase Ib/II of 27%. Increased activity was seen in patients with high *MET*-amplified tumors, with a phase II ORR of 47% in patients with a *MET* gene copy number ≥ 6 [42]. The major difference between these two trials and others is the selection of NSCLC patients with EGFR mutation and MET dysregulation.

### Onartuzumab

A phase II clinical trial compared the treatment of patients with recurrent NSCLC with a combination of onartuzumab and erlotinib (O + E) versus erlotinib alone. Tumor tissue was required to assess MET status by immunohistochemistry (IHC). The study showed no improvement in PFS or OS in the overall population. However, MET-positive patients (*n* = 66) treated with O + E showed improvement in both PFS and OS. Conversely, clinical outcomes were worse in MET-negative patients treated with O + E. Therefore, O + E was associated with improved PFS and OS in the MET-positive population but worse outcomes in MET-negative patients [43]. Further analyses revealed a non-significant OS improvement with O + E in patients with high *MET* copy number (mean ≥ 5 copies/cell by FISH); however, the benefit was maintained in “MET IHC-positive”/MET FISH-negative patients [44]. Based on these findings, the phase III OAM4971g study (METLung) was conducted in 499 patients to examine the efficacy and safety of O + E in patients with locally advanced or metastatic NSCLC selected by MET IHC whose disease had progressed after treatment with a platinum-based chemotherapy regimen. The overall conclusion of this study was that O + E did not improve clinical outcomes, with shorter OS in the onartuzumab arm compared with erlotinib in patients with MET-positive NSCLC [45]. Again, this large validation trial enrolled over 1000 patients but was not conducted in NSCLC patients selected for EGFR mutation/MET dysregulation and relapse from EGFR-TKI treatment.

## Summary and perspective

Osimertinib is now an FDA-approved drug for the treatment of EGFRm NSCLC with T790 mutation after relapse from first- or second-generation EGFR-TKI treatment (second line) and for the therapy of NSCLCs with activating EGFR mutations. However, about 20% of these patients do not respond well to osimertinib. Based on our preclinical findings, NSCLCs with *MET* amplification or protein overexpression/hyperactivation are unlikely to respond to osimertinib or other third-generation EGFRR-TKIs. We predict that most of these non-responders are likely to have *MET* amplification and/or protein hyperactivation. Therefore, it may be necessary to detect MET status before osimertinib treatment. *MET*-amplified EGFRm NSCLCs are likely to be insensitive to osimertinib or other third-generation EGFR-TKIs.

*MET* amplification and MET protein expression are usually detected in the clinic. However, there is no study that detects phosphorylated MET (p-MET), which represents activated MET protein, in EGFRm NSCLC tissues or those with acquired resistance to EGFR-TKIs and its impact on patient response to EGFR-TKIs. In our preclinical studies, *MET*-amplified EGFRm NSCLC cell lines possess not only high levels of MET, but also elevated levels of p-MET [15]. Therefore, the detection of p-MET and its impact as a predictive marker for osimertinib-based therapy against EGFRm NSCLCs should be explored.

In EGFRm NSCLC patients with *MET* amplification and/or hyperactivation or patients relapsed from osimertinib due to *MET* amplification and/or hyperactivation, combinatorial therapy with a MET or MEK inhibitor may be explored based on preclinical and some clinical pilot studies (Fig. 4). Currently, there is an ongoing clinical trial that tests the efficacy of osimertinib in combination with savolitinib in patients with EGFRm+ and MET+, locally advanced or metastatic NSCLC who have progressed following treatment with osimertinib (https://clinicaltrials.gov/ct2/show/NCT03778229) (Table 2). In this trial, MET+ is defined as a high expression of MET (by IHC) and/or increased MET gene copy number (by FISH). We anticipate more similar trials coming in the near future.

The recent development of immunotherapies that target programmed death ligand-1(PD-L1) or programmed death-1 (PD-1) has shown dramatic success in some lung cancer patients [46–48]. However, these immune checkpoint inhibitors were poorly effective in NSCLC patients with EGFR mutations [49]. Recent data show that treatment with MET inhibitors counteracts the induction of PD-1 ligands by interferon-γ in MET-amplified cancers [50]. Whether combining an anti-MET drug with a PD-1 or PD-L1 blockade is a potential strategy against EGFR-mutant NSCLCs relapsed from osimertinib due to *MET* amplification and/or hyperactivation needs further investigation.

## Data Availability

Not applicable as no datasets were generated or analyzed.

## Abbreviations

NSCLC: Non-small cell lung cancer
EGFR: Epidermal growth factor receptor
EGFR-TKIs: EGFR tyrosine kinase inhibitors
